# Antimicrobial resistance mechanisms, treatment challenges, and emerging strategies in *Stenotrophomonas maltophilia* infections

**DOI:** 10.3389/fcimb.2026.1865350

**Published:** 2026-07-27

**Authors:** Siyue Xu, Ziheng Wang, Beibei Li, Yingying Li, Jing Chen, Jie Li, Guangping Xu, Kun Yan, Peng Zhang

**Affiliations:** Department of Laboratory Medicine, The First Affiliated Hospital of Wannan Medical University, Wuhu, Anhui, China

**Keywords:** antimicrobial resistance, bacteriophage therapy, biofilm formation, efflux pumps, emerging therapeutic strategies, *Stenotrophomonas maltophilia*

## Abstract

*Stenotrophomonas maltophilia* is a Gram-negative opportunistic pathogen widely distributed in natural and clinical environments, and has increasingly been recognized as a significant cause of nosocomial infections. Owing to its complex intrinsic resistance mechanisms and the continuous evolution of acquired resistance, the clinical management of *S. maltophilia* infections remains highly challenging. Resistance in *S. maltophilia* is mediated by multiple mechanisms, including chromosomally encoded L1/L2 β-lactamases, multidrug efflux pump systems, reduced outer membrane permeability, and biofilm formation, as well as the acquisition of resistance determinants via mobile genetic elements. In addition, virulence-associated traits, including quorum sensing systems, and extracellular enzyme production, contribute to host adaptation and persistent infection. Trimethoprim–sulfamethoxazole (TMP–SMX) remains the first-line therapy. However, increasing resistance has been reported in certain regions. Alternative and combination therapies, including minocycline and fluoroquinolones, are increasingly utilized. Meanwhile, emerging strategies such as efflux pump inhibitors, anti-virulence approaches, and bacteriophage therapy have attracted growing attention. This review provides a comprehensive overview of the resistance mechanisms, virulence-associated phenotypes, and current therapeutic advances of *S. maltophilia*, with particular emphasis on resistance evolution and novel treatment strategies. These insights may advance our understanding of host–pathogen interactions and support the development of targeted therapeutic approaches for improved clinical management.

## 1 Introduction

The *Stenotrophomonas maltophilia* complex (Smc) is a complex comprising more than 20 closely related species, including *Stenotrophomonas maltophilia*, *Stenotrophomonas pavanii*, and *Stenotrophomonas geniculata (*Li et al., 2023). Genome-based studies have shown that these species cannot be reliably distinguished using routine phenotypic methods or 16S rRNA gene sequencing alone (Yu and Wang, 2024). Although *S. maltophilia* is increasingly recognized as part of Smc, most clinical and antimicrobial susceptibility studies still report isolates as *S. maltophilia* based on conventional laboratory identification. Therefore, this review retains the term *S. maltophilia* to remain consistent with the cited clinical literature, while acknowledging the taxonomic complexity of this organism.

*S. maltophilia* is an opportunistic Gram-negative pathogen, and its prevalence has continued to rise in recent years (Banar et al., 2023; Alfonaisan et al., 2024). This organism exhibits strong ecological adaptability and is widely found in natural environments such as water sources, soil and plant roots. It can also survive on the surface of medical devices for a long time and become a potential source of transmission of infection in the hospital (Brooke, 2012). Infections caused by *S. maltophilia* encompass a wide range of clinical manifestations, including pneumonia, bacteremia, and skin and soft tissue infections (Mojica et al., 2022). However, clinical interpretation can be challenging when *S. maltophilia* is isolated from respiratory specimens or polymicrobial cultures, because positive cultures may reflect colonization rather than true infection and should be interpreted together with clinical, radiological, and microbiological findings, including co-detected pathogens (Pathmanathan and Waterer, 2005). Patients in intensive care units (ICUs), individuals with malignancies, recipients of hematopoietic stem cell transplantation (HSCT) and other immunocompromised populations are considered the primary high-risk hosts (Al-Anazi and Al-Jasser, 2014; Mojica et al., 2022). A meta-analysis reported that the mortality rate associated with *S. maltophilia* bacteremia can approach 40%, with poorer clinical outcomes observed particularly among critically ill and immunocompromised patients (Huang et al., 2024b).

The clinical management of *S. maltophilia* infections is particularly difficult, which stems from its significant inherent drug resistance and continuously evolving acquired drug resistance (Mojica et al., 2022). Owing to its inherent resistance profile, this organism exhibits natural resistance to a wide range of antimicrobial agents, including most β-lactams and aminoglycosides (Chang et al., 2015; Mojica et al., 2022). *S. maltophilia* can also form robust biofilms, which enhance tolerance to antimicrobial agents and host immune defenses, thereby contributing to persistent infection and increasing the risk of treatment failure (Hall and Mah, 2017; Pompilio et al., 2020). Such biofilm-forming capacity is therefore considered a key determinant of its pathogenicity (Pompilio et al., 2020).

TMP–SMX has long been used as the first-line treatment for *S. maltophilia* infections. In recent years, however, resistance to this agent has been increasingly reported in certain regions (Alfonaisan et al., 2024). Alternative agents, including minocycline and levofloxacin, retain activity against a subset of isolates. Their susceptibility profiles, however, vary considerably across regions and may be influenced by epidemiological characteristics, strain backgrounds, and underlying resistance mechanisms (Dadashi et al., 2023).

Despite the increasing number of studies on *S. maltophilia* in recent years, the molecular basis of resistance development, the interplay between resistance and virulence, and the clinical applicability of different therapeutic strategies remain insufficiently integrated. Particularly in the context of limited front-line treatment options, uneven evidence supporting alternative therapies, and immature emerging interventions, it is urgent to conduct a more systematic synthesis and evaluation and evaluation of the resistance mechanisms and therapeutic advances of the pathogen.

Unlike the previous reviews, which mainly focused on a single aspect such as drug resistance mechanisms or virulence, this review provides a comprehensive overview of both intrinsic and acquired resistance mechanisms in *S. maltophilia*. It further summarizes key virulence- and tolerance-associated phenotypes and their relationships with antimicrobial resistance, and systematically evaluates current therapeutic options, emerging intervention strategies and their limitations. This review aims to provide a reference for the precise diagnosis and treatment of *S. maltophilia* infections, as well as for future research.

## 2 Epidemiology and clinical manifestations

### 2.1 Global epidemiology and regional variation

A recent systematic review and meta-analysis reported a global pooled prevalence of *S. maltophilia* isolation from clinical samples of approximately 5.3%, with marked regional variation. Higher estimates were reported in the Western Pacific Region and the European Region, whereas lower estimates were reported in the Region of the Americas (Banar et al., 2023). In China, CARSS/BRICS surveillance data showed that the prevalence of *S. maltophilia* among clinical bacterial isolates remained relatively stable at approximately 2.1% from 2014 to 2021 (Wang et al., 2025). The China-specific estimate appears numerically lower than the global pooled estimate; however, these data should not be directly compared because they were derived from different surveillance systems, study designs, specimen sources and microbiological methods. **Table 1** summarizes regional differences in the reported occurrence of *S. maltophilia* based on published meta-analyses and surveillance studies (Chang et al., 2015; Banar et al., 2023; Wang et al., 2025).

**Table 1:** Regional variation in the reported occurrence of *S. maltophilia* based on a published meta-analysis and surveillance data.

Region	Reported estimate	Source type	Reference
Global	5.3% in the global analysis published in 2023	Systematic review and meta-analysis	(Banar et al., 2023)
Europe	7.9% in the 2023 regional meta-analysis; early European SENTRY data reported *S. maltophilia* among approximately 1.0% of all pathogens	Meta-analysis; international surveillance	(Chang et al., 2015; Banar et al., 2023)
Americas	4.3% in the 2023 regional meta-analysis; early Latin American SENTRY data reported approximately 0.8% among all pathogens	Meta-analysis; international surveillance	(Chang et al., 2015; Banar et al., 2023)
Asia/Western Pacific Region	Asia 7.1% and Western Pacific Region 10.5% in the 2023 meta-analysis	Systematic review and meta-analysis	(Banar et al., 2023)
Oceania	Not separately estimated	Literature limitation	(Banar et al., 2023)
China	Approximately 2.1% of all clinical bacterial isolates from 2014 to 2021	CARSS/BRICS surveillance	(Wang et al., 2025)

The estimates and observations summarized in this table should be interpreted descriptively rather than as directly comparable prevalence estimates.

CARSS, China Antimicrobial Resistance Surveillance System; BRICS, Blood Bacterial Resistant Investigation Collaborative System.

### 2.2 Clinical manifestations, risk factors, and outcomes

**Figure 1:**
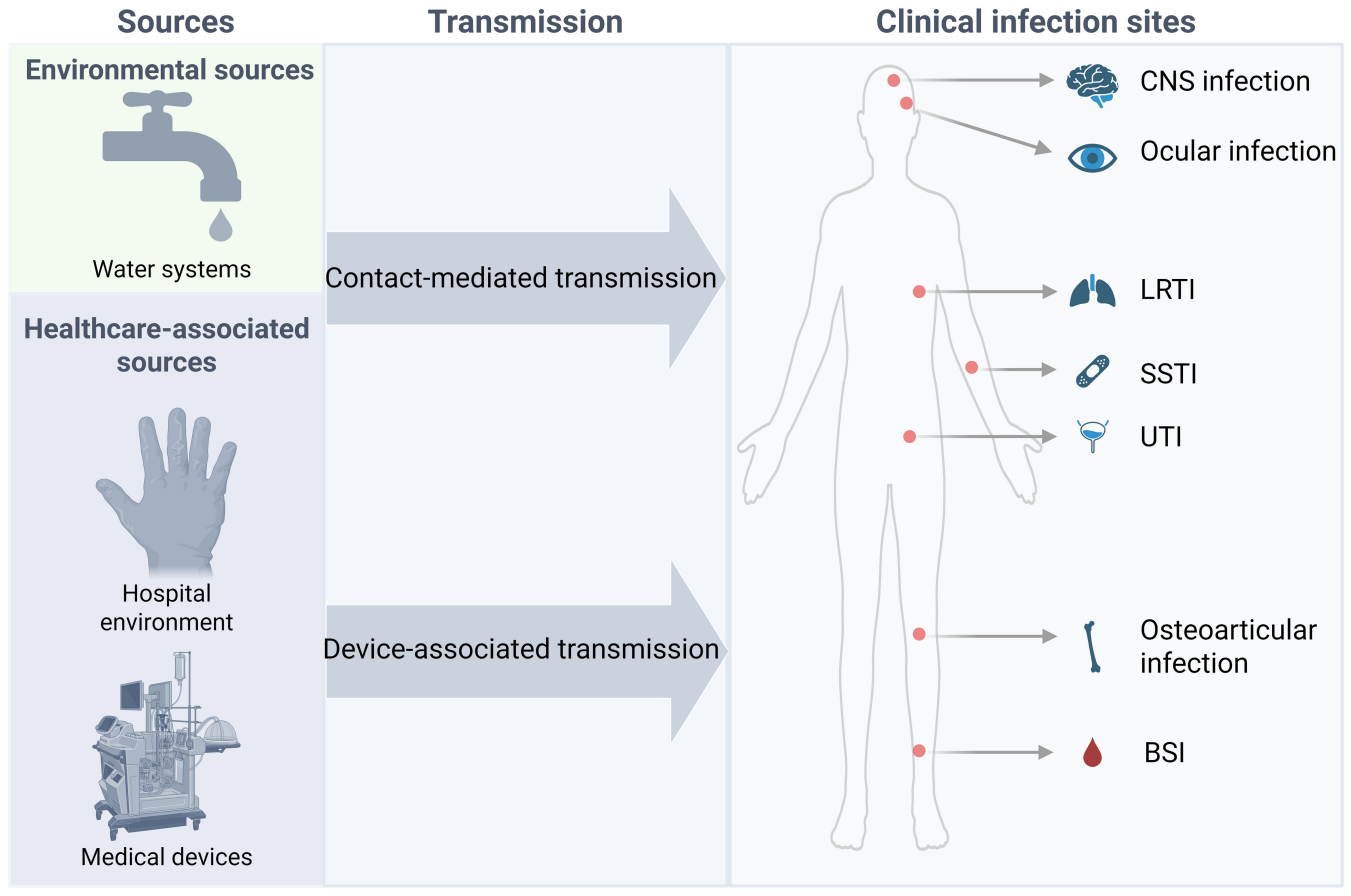
Schematic representation of the sources, transmission pathways, and clinical infection sites of *S. maltophilia*. The schematic summarizes environmental and healthcare-associated reservoirs, contact- and device-associated transmission routes, and major clinical infection sites. CNS, central nervous system; LRTI, lower respiratory tract infection; SSTI, skin and soft tissue infection; UTI, urinary tract infection; BSI, bloodstream infection. Created with BioRender.com.

*S. maltophilia* is widely distributed in environmental and healthcare-associated reservoirs, including water systems, hospital environments, and medical devices, and transmission in healthcare settings may occur through contact-mediated or device-associated routes, particularly in patients exposed to respiratory equipment, indwelling catheters, or other invasive devices (Weber et al., 1999; Guyot et al., 2013; Guy et al., 2016; Brooke, 2021; Cristina et al., 2024). *S. maltophilia* is capable of causing multisystem infections. In addition to common lower respiratory tract infections (LRTIs), bloodstream infections (BSIs), and urinary tract infections (UTIs), it can also involve multiple sites, including skin and soft-tissue infections (SSTIs), the central nervous system (CNS), bones and joints, and the eyes (Brooke, 2021; Hafiz et al., 2022) (**Figure 1**).

Several host- and healthcare-associated factors increase the risk of *S. maltophilia* infection, particularly in critically ill, chronically ill, or immunocompromised patients (Al-Anazi and Al-Jasser, 2014; Mojica et al., 2022). Major risk factors include chronic respiratory disease, cystic fibrosis, malignancy, neutropenia, transplantation, prolonged hospitalization, ICU admission, invasive devices, mechanical ventilation, central venous catheterization, and prior exposure to broad-spectrum antibiotics (Brooke, 2021). The major risk factors and reported outcomes in key patient populations are summarized in **Table 2**.

**Table 2:** Major risk factors and outcomes of *S. maltophilia* infection in key patient populations.

Patient population	Major risk factors	Reported outcomes	References
Hospitalized or ICU patients	ICU admission, mechanical ventilation, invasive devices, prior broad-spectrum antibiotics	High short-term mortality, especially in severe pneumonia or bloodstream infection	(Brooke, 2021; Wang et al., 2021)
Bloodstream infection patients	Central venous catheterization, malignancy, neutropenia, septic shock, inappropriate antimicrobial therapy	30-day mortality of 46.1% reported in a multicenter study	(Gezer et al., 2024; Huang et al., 2024b)
Cystic fibrosis patients	Chronic lung disease, impaired airway clearance, repeated antibiotic exposure, polymicrobial airway colonization	Clinical impact varies according to lung function, co-infection, and disease severity	(Terlizzi et al., 2023)
Cancer or hematologic malignancy patients	Solid tumors, hematologic malignancy, chemotherapy, neutropenia, mucosal barrier injury	High mortality reported, particularly with bacteremia or hemorrhagic pneumonia	(Aisenberg et al., 2007; Huang et al., 2024a; Guo W. et al., 2025)
Transplant recipients	Immunosuppression, transplantation, invasive procedures, central venous catheterization, broad-spectrum antibiotics	Increased risk of invasive infection; outcomes vary by transplant type and infection severity	(Al-Anazi and Al-Jasser, 2014)

Reported outcomes vary across studies because of differences in patient populations, infection sites, disease severity, definitions of infection versus colonization, and follow-up periods.

ICU, intensive care unit.

Overall, the clinical significance and prognosis of *S. maltophilia* infection depend on the patient population, infection site, severity of illness and immune status.

## 3 Mechanisms of antimicrobial resistance

### 3.1 Antimicrobial susceptibility and resistance trends

**Figure 2:**
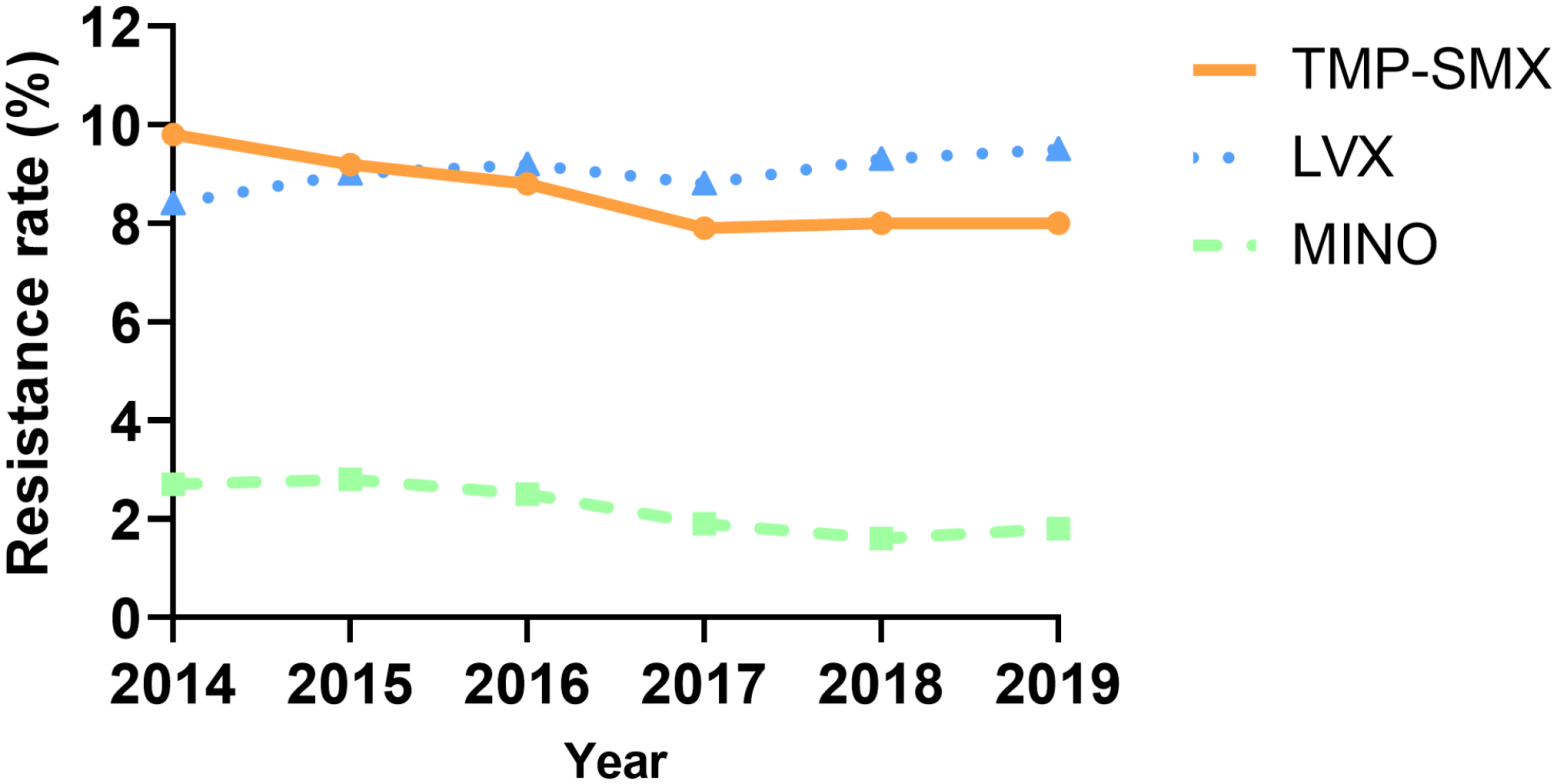
China-specific trends in antimicrobial resistance of *S. maltophilia* to TMP-SMX, levofloxacin, and minocycline from 2014 to 2019 based on CARSS surveillance data. The figure shows observed annual resistance rates reported by the China Antimicrobial Resistance Surveillance System (CARSS) (China Antimicrobial Resistance Surveillance System, 2021). TMP-SMX, trimethoprim-sulfamethoxazole; LVX, levofloxacin; MINO, minocycline.

According to China-specific CARSS surveillance data, the resistance rates of *S. maltophilia* to TMP–SMX, levofloxacin, and minocycline remained relatively low from 2014 to 2019 (China Antimicrobial Resistance Surveillance System, 2021). TMP–SMX resistance ranged from 7.9% to 9.8%, levofloxacin resistance ranged from 8.4% to 9.5%, and minocycline resistance ranged from 1.6% to 2.8% (**Figure 2**). A subsequent CARSS/BRICS-based national analysis further reported that TMP–SMX resistance decreased to 7.5% in 2021, levofloxacin resistance did not exceed 9.5%, and minocycline resistance remained below 3% throughout 2014–2021 (Wang et al., 2025).

International surveillance studies also suggest geographic variation in the antimicrobial susceptibility of *S. maltophilia*. In a global analysis of 1, 586 clinical isolates collected from the Asia-Pacific region, Europe, Latin America, and North America, levofloxacin susceptibility varied geographically, with a relatively higher susceptibility rate reported in Latin America than in several other regions (Farrell et al., 2010). These data should be interpreted descriptively because the underlying studies differed in surveillance periods, geographic coverage, isolate sources, susceptibility testing methods, and breakpoint criteria.

### 3.2 Intrinsic resistance mechanisms

The intrinsic resistance mechanisms of *S. maltophilia* are complex and are primarily associated with chromosomally encoded β-lactamases, multidrug efflux pumps, and reduced outer membrane permeability (Sánchez, 2015).

The limited efficacy of β-lactam antibiotics is a defining feature of the intrinsic resistance of *S. maltophilia*. This resistance is mainly driven by the synergistic activity of two chromosomally encoded β-lactamases, L1 and L2 (Mojica et al., 2019). L1 is a zinc-dependent metallo-β-lactamase with the ability to hydrolyze a wide range of β-lactam antibiotics, including carbapenems. By comparison, L2 belongs to class A serine cephalosporinases and can be inhibited by clavulanic acid and avibactam (Wang et al., 2018). Although L1 and L2 are often described together, previous studies have shown that they differ in genetic organization and AmpR-mediated regulation. The gene encoding L2 β-lactamase is located adjacent to the divergently transcribed regulatory gene *ampR*, forming an AmpR–L2 regulatory module. By contrast, the gene encoding L1 β-lactamase is not directly linked to a neighboring ampR-like regulatory gene. AmpR is required for the inducible expression of both β-lactamases in response to β-lactam exposure; however, in the absence of an inducer, AmpR has been reported to activate basal L1 expression while repressing L2 expression. Under β-lactam-induced conditions, *AmpR* can activate the expression of both L1 and L2 β-lactamases, indicating that these two β-lactamases are coordinately regulated but not controlled in an identical manner (Lin et al., 2009).

Multidrug efflux pump systems play a central role in the intrinsic resistance of *S. maltophilia*. Among them, resistance–nodulation–division (RND) family efflux pumps are the most prominent. SmeDEF is one of the important RND efflux systems in this organism. It can reduce the intracellular accumulation of a variety of antibacterial drugs such as fluoroquinolones, tetracyclines, macrolides and TMP-SMX in cells, thus broadening its resistance spectrum (Zhang et al., 2001; Sánchez and Martínez, 2015). Its expression is mainly controlled by the local repressor SmeT, and mutations or functional alterations in this regulator may lead to SmeDEF overexpression and multidrug resistance (Sánchez et al., 2002). As another member of the RND family, SmeABC also plays an important role in antimicrobial resistance of *S. maltophilia*. Previous studies have demonstrated that overexpression of SmeABC significantly increases resistance to ciprofloxacin (Chang et al., 2004). In multidrug-resistant clinical isolates, co-overexpression of SmeABC and SmeDEF has been observed, suggesting potential synergistic contributions among different efflux systems (Cho et al., 2012). In addition to SmeDEF and SmeABC, the SmeVWX system has been associated with multidrug resistance. Its overexpression represents an important mechanism underlying resistance to TMP–SMX and fluoroquinolones in *S. maltophilia (*Sánchez and Martínez, 2018). Notably, vitamin K3 has been shown to induce the expression of the SmeVWX efflux pump, thereby enhancing bacterial multidrug resistance (Blanco et al., 2017). SmeYZ is a constitutively expressed RND efflux pump that contributes to aminoglycoside resistance and may also be linked to bacterial physiology and virulence-associated phenotypes (Lin et al., 2015). SmeIJK and SmeOP have also been implicated in reduced susceptibility to multiple antimicrobial classes, including aminoglycosides, tetracyclines, quinolones, and macrolides (Huang et al., 2014; Lin et al., 2014). Beyond RND pumps, non-RND transporters such as the ATP-binding cassette (ABC) transporter SmrA and other ABC-type efflux systems may contribute to fluoroquinolone resistance or stress adaptation (Al-Hamad et al., 2009). Together, these findings indicate that multidrug resistance in *S. maltophilia* reflects the combined contribution of multiple RND and non-RND transport systems rather than the activity of a single dominant pump.

Reduced outer membrane permeability and altered outer membrane protein function also contribute to antimicrobial resistance and bacterial fitness in *S. maltophilia*. OmpA is an abundant outer membrane protein with both channel-related and structural functions. In an OmpA C-terminal truncation mutant, the most evident susceptibility changes involved β-lactam antibiotics, with marked decreases in the minimum inhibitory concentrations (MICs) of ceftazidime and ticarcillin–clavulanate, whereas the effects on quinolones and TMP–SMX were mild or not significant (Li et al., 2022).

Classical porins associated with the influx of carbapenems and other antimicrobial agents include OmpK35 and OmpK36 in *Klebsiella pneumoniae*, OmpF in *Serratia marcescens*, and the carbapenem-associated substrate-specific channel OprD in *Pseudomonas aeruginosa (*Mammeri et al., 2025). In an experimental evolution study using Escherichia coli, Knopp and Andersson showed that deletion of the two major porins OmpC and OmpF increased carbapenem resistance but imposed a substantial fitness cost. After serial passage, compensatory mutations upregulated alternative porins, including PhoE and ChiP, thereby improving bacterial fitness. Notably, PhoE overexpression restored carbapenem susceptibility, whereas ChiP overexpression compensated for the fitness cost while maintaining the carbapenem-resistant phenotype (Knopp and Andersson, 2015).

### 3.3 Acquired resistance

Acquired resistance in *S. maltophilia* refers to additional resistance phenotypes that arise through genetic mutations, alterations in regulatory mechanisms, or the acquisition of exogenous resistance genes.

Mobile genetic elements, including integrons, transposons, insertion sequence-related elements, plasmids, and genomic islands, contribute to acquired resistance in *S. maltophilia* by facilitating the acquisition and dissemination of antimicrobial resistance determinants (Wang et al., 2018). Integrons primarily function as gene-capture and expression platforms, whereas their dissemination usually depends on their association with mobile genetic elements such as plasmids, transposons, insertion sequences, or integrative and conjugative elements (Gillings, 2014; Partridge et al., 2018). By contrast, transposons are mobile DNA elements that can move between genomic locations and may carry resistance genes, integrons, or both (Partridge et al., 2018).

Class 1, class 2, and class 3 integrons are mainly distinguished by their integrase genes, *intI1*, *intI2*, and *intI3*, respectively, and by their genetic contexts and cassette repertoires (Deng et al., 2015). Class 1 integrons commonly contain a 5′ conserved segment with intI1 and often a 3′ conserved segment carrying qacEΔ1 and sul1. In *S. maltophilia*, class 1 integrons have been associated with sul1 and dfrA genes related to TMP–SMX resistance, as well as aminoglycoside resistance genes such as aadA/ant(3″)-Ia, aacA4, and aac(6′)-Ib. Class 2 integrons, typically associated with Tn7-family transposons, usually carry a more restricted cassette array such as *dfrA1*, *sat2*, and *aadA1*, which are related to trimethoprim, streptothricin, and streptomycin resistance, respectively. Class 3 integrons are much rarer; although they can carry resistance gene cassettes, their occurrence and resistance-gene content in *S. maltophilia* remain poorly characterized compared with class 1 integrons (Deng et al., 2015; Fonseca and Vicente, 2022).

Different class 1 integron-associated resistance genes confer resistance to different antimicrobial classes. sul1 encodes an alternative dihydropteroate synthase and is associated with sulfonamide resistance, whereas *dfrA* genes encode trimethoprim-resistant dihydrofolate reductase variants. Aminoglycoside resistance gene cassettes, including aadA/ant(3”)-Ia, aacA4, and aac(6’)-Ib, encode aminoglycoside-modifying enzymes and are associated with resistance to selected aminoglycosides (Ramirez et al., 2013; Deng et al., 2015; Hu et al., 2016; Wang et al., 2018).

Transposon- or transposase-associated resistance contexts have also been reported in *S. maltophilia*. Reported transposon-related contexts include Tn1/Tn3-type or Tn3-family transposons, ISCR-associated elements, IS26-flanked composite transposon structures, and transposase-linked *sul2* loci (Toleman et al., 2007; He et al., 2015; Wang et al., 2018; Boncompagni et al., 2025). Resistance determinants identified in these contexts include *blaTEM-2*, *sul2*, *floR*, *tet(A)-tetR*, *strA/strB*, *sul1*, *aadA2*, *blaVIM-1*, *aac(6′)-Ib*, *aac(6′)-31*, and *qacEΔ1*, depending on the specific element and isolate (Avison et al., 2000; Toleman et al., 2007; He et al., 2015; Boncompagni et al., 2025). These examples indicate that transposons and related mobilizable elements can contribute to the acquisition and dissemination of resistance determinants in *S. maltophilia*, but their prevalence and clinical impact remain incompletely defined.

The mobile sulfonamide resistance genes *sul3* and *sul4* should be discussed cautiously in *S. maltophilia*. The *sul3* gene has been reported on conjugative plasmids or atypical class 1 integron-related structures in other bacteria (Perreten and Boerlin, 2003; Antunes et al., 2007; Wyrsch et al., 2022). The *sul4* gene, the fourth described mobile sulfonamide resistance gene, has been reported in association with mobile genetic elements and has recently been detected in clinical isolates of other bacterial species (Razavi et al., 2017; Peng et al., 2023). However, evidence regarding *sul3* and *sul4* in *S. maltophilia* remains limited, and further studies are required to determine whether these genes contribute substantially to TMP–SMX resistance in this species complex.

Plasmid-mediated resistance has been described in *S. maltophilia*, but it appears to be less commonly documented than integron-, transposon-, ISCR-associated, or chromosomal regulatory mechanisms. A previous study reported the presence of resistance genes, including *blaPER*, *sul1*, and *sul2*, as well as class 1 integrons, on IncA/C-type plasmids in *S. maltophilia (*Furlan and Stehling, 2018). Although this finding was identified in environmental isolates, it highlights the potential for plasmid-mediated dissemination of resistance determinants. In addition, resistance genes such as *sul1*, *sul2*, and *dfrA* may be located within plasmid-associated class 1 integrons or other mobile genetic elements, and are closely associated with resistance to TMP–SMX (Hu et al., 2011; Mendes et al., 2020).

**Figure 3:**
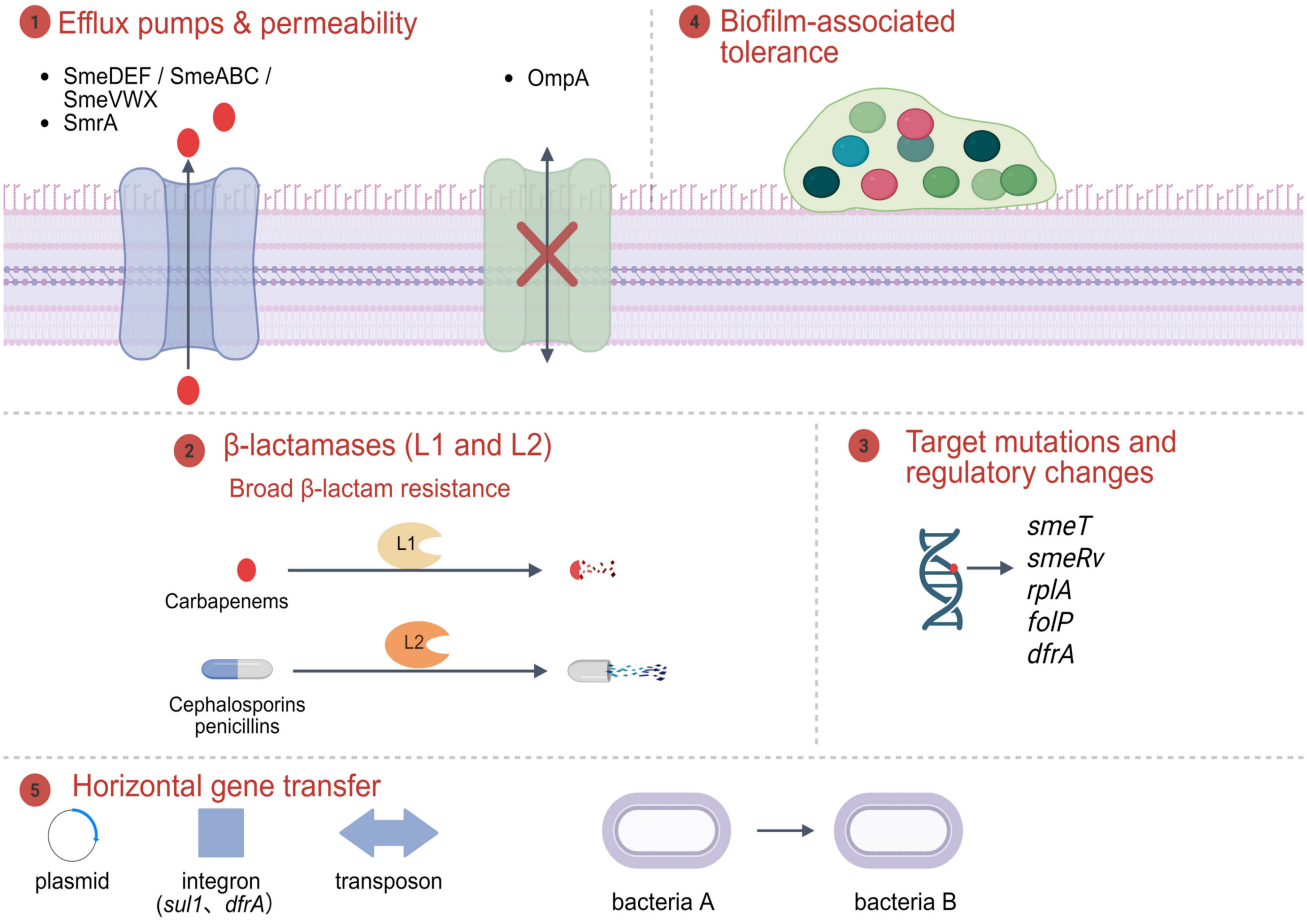
Major mechanisms of antimicrobial resistance in *S. maltophilia*. The figure integrates intrinsic resistance, acquired resistance, reduced permeability, efflux activity, β-lactamase-mediated resistance, and biofilm-associated tolerance. Biofilm formation is interpreted as a contributor to tolerance and persistence rather than stable genetic resistance. Created with BioRender.com.

In addition to mobile genetic elements, mutations in chromosomal resistance-associated regulatory genes also represent an important source of acquired resistance in *S. maltophilia (*Gil-Gil et al., 2020). A typical example is mutations in the *smeT* gene, which can lead to overexpression of the SmeDEF efflux pump, thereby reducing susceptibility to multiple antimicrobial agents, including fluoroquinolones and tetracyclines (Pak et al., 2015). Previous studies have shown that mutations in the *rplA* gene in *S. maltophilia* may be associated with overexpression of the SmeYZ efflux pump, thereby promoting resistance to aminoglycoside antibiotics (Calvopiña et al., 2020). The *folP* gene encodes dihydropteroate synthase, the target of sulfonamides, and alterations in this gene may affect susceptibility to sulfamethoxazole (Huang et al., 2018). In addition, acquired *dfrA* genes encode trimethoprim-resistant dihydrofolate reductase variants and have been associated with TMP–SMX resistance in *S. maltophilia (*Hu et al., 2011). Furthermore, mutations in the efflux pump regulatory gene *smeRv* may affect SmeVWX efflux pump expression and are associated with resistance to TMP–SMX and quinolones (Boonyong et al., 2025). The *rpoN* gene, which encodes the alternative sigma factor RpoN, is mainly associated with flagellar synthesis, swimming motility, and possibly conjugation- or colonization-related phenotypes, rather than being an established acquired resistance determinant in *S. maltophilia (*Liao et al., 2021). A deeper understanding of these molecular mechanisms is crucial for optimizing antimicrobial therapy and controlling the spread of resistant strains (**Figure 3**).

### 3.4 Biofilm-associated antimicrobial tolerance

In addition to intrinsic and acquired resistance mechanisms, *S. maltophilia* can exhibit biofilm-associated antimicrobial tolerance, a phenotypic and adaptive state that differs from stable genetically encoded resistance (Hall and Mah, 2017; Pompilio et al., 2020). Biofilms are complex microbial communities embedded in extracellular polymeric substances, which can restrict antimicrobial penetration and alter bacterial metabolic states, thereby enhancing tolerance to antimicrobial exposure (Idrees et al., 2021). Biofilm growth can increase tolerance to TMP–SMX and levofloxacin, which may contribute to persistent infection and reduced antimicrobial efficacy (Pompilio et al., 2020). *In vitro* studies have shown that low concentrations of moxifloxacin can influence biofilm formation in this organism (Wang et al., 2016). This effect may be related to alterations in bacterial physiological activity. However, these findings mainly reflect biofilm-associated adaptive tolerance under experimental conditions, and their clinical translatability remains to be further validated.

## 4 Virulence-associated phenotypes and pathogenic mechanisms

The virulence mechanisms of *S. maltophilia* are highly complex and adaptable. Although this organism is generally regarded as a low-virulence pathogen, it can still cause serious, and occasionally life-threatening infections (Mikhailovich et al., 2024). This is particularly evident in high-risk populations, including patients with cystic fibrosis, hematological malignancies, prolonged exposure to broad-spectrum antibiotics, or those receiving mechanical ventilation in ICUs (Al-Anazi and Al-Jasser, 2014; Mojica et al., 2022). The pathogenicity of *S. maltophilia* is not driven by a single potent toxin. Instead, it arises from a combination of virulence-associated phenotypes, secreted factors, and regulatory networks that collectively promote bacterial colonization, tissue damage, and persistent infection (Kalidasan et al., 2018; Mojica et al., 2022).

### 4.1 Biofilm formation and persistent colonization

Biofilm formation represents a key virulence-associated phenotype in *S. maltophilia*. Biofilms not only enhance bacterial tolerance to antimicrobial exposure and phagocytic clearance, but also facilitate persistent adhesion and colonization on host surfaces; therefore, they are closely related to chronic infection and treatment failure (Idrees et al., 2021). In healthcare settings, biofilm formation is particularly important for the persistence and transmission of *S. maltophilia* organisms (Brooke, 2021). Biofilm-associated growth on water-related hospital reservoirs, sinks, faucets, respiratory equipment, catheters, and other medical devices may provide a source for repeated contamination and contact- or device-associated transmission (Guyot et al., 2013; Guy et al., 2016).

Although the extent of biofilm formation varies among *S. maltophilia* strains, the ability to form biofilms appears to be a common and relatively conserved phenotype in this species. Clinical and hospital infection-associated isolates often exhibit strong biofilm-forming capacity (Pompilio et al., 2020). In the ANSELM prospective multicenter study, most clinical isolates were able to form biofilms, but biofilm biomass differed across isolates and clinical contexts. Bloodborne isolates, hospital-acquired infection-associated isolates, and isolates classified as definite pathogens tended to show stronger biofilm-forming capacity, and biofilm formation efficiency was positively associated with mechanical ventilation. Mature biofilms also showed markedly reduced susceptibility to commonly used agents, including cotrimoxazole and levofloxacin, compared with planktonic cells (Pompilio et al., 2020). However, a direct and consistent relationship between strong *in vitro* biofilm formation and infection severity or mortality has not been fully established, and larger studies are needed to clarify this association.

Several genes have been implicated in *S. maltophilia* biofilm formation, including *smf-1*, *rmlA*, *rmlC*, *xanB*, *spgM*, and *rpfF* (Huang et al., 2006; García et al., 2022). Among these, *smf-1* is associated with fimbrial adhesion and may contribute to initial attachment to host tissues and abiotic surfaces (de Oliveira-Garcia et al., 2003). The lipopolysaccharide- and exopolysaccharide-related genes *rmlA*, *rmlC*, and *xanB* are involved in surface polysaccharide biosynthesis and influence cell-surface architecture, twitching motility, and biofilm formation (Huang et al., 2006). The *spgM* gene is also involved in polysaccharide-related pathways, whereas rpfF contributes to diffusible signal factor-mediated quorum sensing, collectively influencing biofilm formation and persistence in *S. maltophilia* (Zhuo et al., 2014; Alcaraz et al., 2019). However, no specific allelic variants have been consistently associated with increased biofilm biomass.

### 4.2 Quorum sensing and its regulation of virulence phenotypes

Quorum sensing (QS) is one of the core mechanisms regulating virulence-associated phenotypes in *S. maltophilia (*Bhaumik et al., 2023). QS in this organism is primarily mediated by diffusible signal factor (DSF), also known as cis-11-methyl-2-dodecenoic acid, a diffusible fatty acid signal molecule. DSF is synthesized by RpfF and sensed through the RpfC/RpfG two-component regulatory system (Huedo et al., 2015; Huedo et al., 2018). Importantly, DSF-mediated signaling is not restricted to intraspecies communication. After being secreted into the extracellular environment, DSF can diffuse across bacterial communities and may be perceived by other bacterial species carrying compatible DSF-responsive sensor systems, thereby mediating interspecies communication in polymicrobial communities (Ryan et al., 2008; An et al., 2019). For example, DSF produced by *S. maltophilia* can be sensed by *Pseudomonas aeruginosa* through the PA1396 histidine kinase, leading to altered biofilm formation and increased polymyxin tolerance (Ryan et al., 2008; An et al., 2019). This interspecies signaling may have clinical relevance in mixed infections involving *S. maltophilia* and *P. aeruginosa*, two opportunistic pathogens frequently associated with hospital-acquired infections. Targeting QS has therefore been proposed as a promising anti-virulence strategy. This approach aims to attenuate pathogenic phenotypes rather than directly inhibit bacterial growth, thereby theoretically reducing the selective pressure for the emergence and enrichment of resistant strains (Guillén-Navarro et al., 2023).

### 4.3 Extracellular enzymes and secretion systems

*S. maltophilia* secretes a variety of extracellular enzymes, which can cause damage to the host tissues and impair local host defense mechanisms, among which the protease StmPr1 is particularly important (Strateva et al., 2023). StmPr1 has been identified as one of the major secreted serine proteases of *S. maltophilia*. This enzyme catalyzes protein hydrolysis and degrades extracellular matrix components such as collagen, thereby aggravating host cell damage and facilitating bacterial invasion (Sommer et al., 2025). In addition to StmPr1, other extracellular proteases, including the serine proteases StmPr2 and StmPr3, may also contribute to protease-mediated pathogenicity (DuMont et al., 2015; Molloy et al., 2019). StmPr1 and StmPr2 have been shown to account for type II secretion system-dependent serine protease, gelatinase, and caseinolytic activities, whereas StmPr3 has also been identified as a major extracellular serine protease that may participate in the degradation of pulmonary antiproteases and innate immune dysfunction (DuMont et al., 2015; Molloy et al., 2019; Fluit et al., 2022).

*S. maltophilia* harbors multiple protein secretion systems capable of delivering effector molecules to the extracellular environment or to neighboring cells. In addition to the classical type II secretion system (T2SS), recent studies have highlighted the role of the type IV secretion system (T4SS). The T4SS mediates contact-dependent interbacterial killing and can suppress the growth of competing organisms, including *Pseudomonas aeruginosa* isolated in clinical settings (Nas et al., 2021).

### 4.4 Other virulence-associated factors

Beyond extracellular enzymes and secretion systems, several other virulence-associated factors contribute to the pathogenic potential of *S. maltophilia*. Clinical strains of *S. maltophilia* can produce flagella, which contribute to bacterial motility and adhesion (de Oliveira-Garcia et al., 2002). The major flagellar structural protein FliC has been shown to be immunogenic and is also associated with bacterial adhesion (Zgair and Chhibber, 2011). Iron acquisition systems, including siderophore- and heme-mediated uptake pathways, also support bacterial survival under iron-limited host conditions and may influence biofilm formation, oxidative stress responses, extracellular enzyme production, and other virulence-associated phenotypes (García et al., 2015; Kalidasan et al., 2018). Adhesins, including fimbriae and pili such as Smf-1, facilitate attachment to epithelial cells and abiotic surfaces and contribute to biofilm initiation and persistence (de Oliveira-Garcia et al., 2003; Bhaumik et al., 2023).

## 5 Clinical treatment challenges and current therapeutic options

### 5.1 Conventional antimicrobial agents

Recent updates to the Clinical and Laboratory Standards Institute (CLSI) M100 guidelines have revised antimicrobial susceptibility breakpoints for *S. maltophilia*. The 2024 CLSI update removed interpretive breakpoints for ceftazidime and tightened those for minocycline, whereas the current CLSI M100-Ed35 (2025) retains the updated interpretive criteria for *S. maltophilia* (Bakthavatchalam et al., 2024; Clinical and Laboratory Standards Institute, 2025) (**Table 3**). These revisions are primarily based on pharmacokinetic/pharmacodynamic (PK/PD) evidence and clinical efficacy data. Their purpose is to improve the interpretation of susceptibility testing results, rather than to reflect actual changes in resistance levels among clinical isolates.

**Table 3:** Antimicrobial agents for *S. maltophilia*: conventional and emerging therapeutic options.

Category	Agent	Clinical application	Susceptibility (%)	CLSI breakpoint(S, μg/mL)	References
Conventional agents	TMP-SMX	First-line therapy	84–97	≤2/38	(Tamma et al., 2022a; Lee et al., 2023; Clinical and Laboratory Standards Institute, 2025; Guo Y. et al., 2025; Sader et al., 2025)
	Minocycline	Alternative therapy	70–90 (previous)/35–70 (updated)	≤1	(Bakthavatchalam et al., 2024; Hevia et al., 2024; Clinical and Laboratory Standards Institute, 2025; Graves et al., 2025; Sader et al., 2025)
	Levofloxacin	Alternative therapy	50–80	≤2	(Lee et al., 2023; Sader et al., 2025)
Emerging agents	Cefiderocol	Alternative therapy	>95	≤1	(Karlowsky et al., 2022; Karakonstantis et al., 2024; Clinical and Laboratory Standards Institute, 2025)
	Eravacycline	Alternative therapy	N/A	No CLSI breakpoint available	(Morrissey et al., 2020; Wu et al., 2023)
	Omadacycline	Alternative therapy	N/A	No CLSI breakpoint available	(Xiao et al., 2020)

TMP–SMX, trimethoprim–sulfamethoxazole; N/A, not available.

CLSI breakpoints are presented according to CLSI M100-Ed35(2025). The value “≤2/38” represents the susceptible breakpoint for TMP–SMX. “Previous” and “updated” indicate different breakpoint criteria based on earlier and revised CLSI standards.

TMP–SMX has long served as the first-line conventional agent for the treatment of *S. maltophilia* infections. It retains high activity against most *S. maltophilia* isolates, which further supports its continued role in the management of these infections (Tamma et al., 2022a). Multicenter surveillance data indicate that the susceptibility of *S. maltophilia* to TMP–SMX has been reported to range from 84% to 97%, based on isolates collected from the United States during 2019–2023 and from the ATLAS program during 2004–2020 (Lee et al., 2023; Sader et al., 2025). Data from the CHINET 2024 surveillance report showed that, among clinical *S. maltophilia* isolates collected from hospitals across China in 2024, the TMP–SMX resistance rate was approximately 6.5%, while the susceptibility rate exceeded 90% (Guo Y. et al., 2025). These findings indicate that TMP–SMX remains highly active against *S. maltophilia* in this setting.

When TMP-SMX resistance develops or TMP-SMX cannot be used, alternative regimens should be guided by susceptibility testing and current clinical guidance. The 2024 IDSA guidance supports combination therapy using two active agents selected from cefiderocol, minocycline, TMP-SMX, and levofloxacin, or the combination of ceftazidime-avibactam plus aztreonam. Aztreonam-avibactam has shown potent *in vitro* activity against *S. maltophilia* and may represent an additional option where available, although clinical data remain limited (Sader et al., 2020; Tamma et al., 2024). Among these options, minocycline shows relatively high *in vitro* activity against *S. maltophilia*. Surveillance studies, including isolates from United States medical centers during 2019–2023 and the ATLAS program during 2004–2020, have reported relatively high minocycline susceptibility; however, susceptibility estimates may decrease when revised CLSI minocycline breakpoints are applied (Lee et al., 2023; Bakthavatchalam et al., 2024; Sader et al., 2025). Fluoroquinolones, including levofloxacin, retain activity against a subset of isolates. Reported susceptibility rates, however, vary considerably across studies and typically fall within the range of 50%–80% (Lee et al., 2023; Sader et al., 2025). This variability may limit their reliability in clinical use. Doxycycline, another tetracycline-class agent, has been used to treat ventilator-associated pneumonia caused by *S. maltophilia* in a case report (Wood et al., 2010). Small retrospective studies indicate that its clinical efficacy may be comparable to that of TMP–SMX (Alhayani et al., 2024). However, the overall clinical evidence remains limited. The management of *S. maltophilia* infections should extend beyond *in vitro* antimicrobial activity alone. Therapeutic decisions must take into account antimicrobial susceptibility results, the site of infection, tissue penetration, host factors, and source control (Tamma et al., 2024).

### 5.2 Emerging antimicrobial agents

#### 5.2.1 Cefiderocol

Cefiderocol is a novel siderophore cephalosporin that enters bacterial cells via iron transport systems. It exhibits *in vitro* activity against a broad range of multidrug-resistant Gram-negative pathogens, highlighting its potential as a promising therapeutic option (Shortridge et al., 2022). The use of cefiderocol in the treatment of *S. maltophilia* infections has received increasing attention in recent years (Nakamura et al., 2021). The 2022 Infectious Diseases Society of America (IDSA) guidelines for the management of antimicrobial-resistant Gram-negative infections recommend the use of cefiderocol as part of combination therapy for *S. maltophilia* infections (Tamma et al., 2022b). This approach is particularly recommended for moderate to severe cases. In such situations, cefiderocol is typically administered in combination with other active agents until clinical improvement is achieved (Tamma et al., 2022b). *In vitro* susceptibility data, including SIDERO-WT surveillance studies from 2014 to 2019, indicate that cefiderocol demonstrates high activity against *S. maltophilia*, with susceptibility rates exceeding 95% among clinical isolates (Karlowsky et al., 2022; Karakonstantis et al., 2024). CLSI M100-Ed35 (2025) defines the susceptible-only breakpoint for cefiderocol against *S. maltophilia* as a MIC of ≤1 μg/mL (Clinical and Laboratory Standards Institute, 2025). Cefiderocol is able to overcome several intrinsic resistance mechanisms of *S. maltophilia*. These include the production of L1 metallo-β-lactamase and reduced outer membrane permeability (Yamano, 2019). This makes it a promising therapeutic option for these infections. In recent years, multiple cases have reported successful treatment of *S. maltophilia* infection using cefiderocol either as monotherapy or in combination regimens (Chambers et al., 2023; Hsu et al., 2023; Medioli et al., 2023). Overall, cefiderocol represents a potential therapeutic option when conventional antimicrobial therapies fail or when infections are caused by multidrug-resistant strains, although clinical evidence remains limited (Karlowsky et al., 2022; Tamma et al., 2022b; Karakonstantis et al., 2024).

#### 5.2.2 Eravacycline

Eravacycline is a novel tetracycline-class antimicrobial agent with potential activity against *S. maltophilia*. *In vitro* studies have demonstrated that eravacycline retains potent antimicrobial activity against *S. maltophilia* isolates resistant to TMP–SMX or levofloxacin, with a minimum inhibitory concentration required to inhibit 90% of isolates (MIC_90_) of approximately 2 μg/mL (Morrissey et al., 2020; Wu et al., 2023). These findings suggest potential utility against multidrug-resistant strains. Clinical evidence, however, remains limited, and its efficacy in real-world settings is still uncertain (Al Musawa et al., 2025). At present, clinical guidelines do not recommend eravacycline as a standard therapeutic option (Tamma et al., 2024). Further high-quality studies are needed to validate its clinical efficacy and safety.

#### 5.2.3 Omadacycline

Omadacycline, another novel tetracycline-class antimicrobial agent, has received increasing attention for its potential use in the treatment of *S. maltophilia* infections. In a multicenter Chinese study of bacterial isolates collected from 2014 to 2017, omadacycline showed moderate *in vitro* activity against *S. maltophilia*, with a minimum inhibitory concentration required to inhibit 90% of isolates (MIC_90_) of approximately 4 μg/mL (Xiao et al., 2020). A separate study evaluated isolates resistant to TMP–SMX or levofloxacin and found that omadacycline retains inhibitory activity against a subset of resistant strains. However, its overall antimicrobial activity appears to be lower than that of minocycline (Biagi et al., 2020). Accordingly, current clinical guidelines have not yet recommended omadacycline as a standard therapeutic option for *S. maltophilia* infections, and further *in vivo* studies and clinical trials are required to establish its efficacy (Tamma et al., 2024).

### 5.3 Combination therapy and synergistic strategies

With the increasing antimicrobial resistance of *S. maltophilia*, combination antimicrobial therapy has gradually emerged as an important strategy to improve therapeutic efficacy.

According to guidelines from the Infectious Diseases Society of America, combination therapy with two antimicrobial agents demonstrating *in vitro* activity is generally recommended for moderate-to-severe *S. maltophilia* infections, in order to improve treatment success rates and reduce the emergence of resistance (Tamma et al., 2022b; Tamma et al., 2024). *In vitro* studies suggest that TMP–SMX monotherapy may exhibit limited bactericidal activity against certain isolates, whereas combination regimens with agents such as ciprofloxacin or tobramycin demonstrate enhanced bactericidal effects (Zelenitsky et al., 2005). A study by Hu L-F et al. reported that TMP–SMX combined with ceftazidime, as well as levofloxacin combined with ceftazidime, exhibited strong synergistic antimicrobial activity *in vitro (*Hu et al., 2014). The combination of moxifloxacin with ticarcillin/clavulanate has shown bactericidal activity *in vitro* and appears to reduce the emergence of resistant subpopulations, potentially providing a therapeutic option for immunocompromised or critically ill patients (Zhao et al., 2022). In addition, tigecycline in combination with colistin has been reported in isolated cases of severe infections; however, current evidence remains limited to case reports, and its efficacy and safety have not yet been supported by high-quality studies (Church et al., 2013). Overall, clinical evidence supporting some alternative agents remains limited, and further prospective studies are required to validate their efficacy and safety. Therefore, antimicrobial therapy should be guided by local resistance patterns and antimicrobial susceptibility testing results to ensure optimal clinical outcomes.

Representative real-world evidence for different *S. maltophilia* infection types is summarized in **Table 4**. Because most available data are derived from retrospective studies, case series, or case reports, these examples should be interpreted as illustrative clinical experience rather than definitive comparative efficacy evidence.

**Table 4:** Representative real-world evidence for treatment of *S. maltophilia* infections.

Infection type	Treatment regimen	Outcome	Reference
Hospital-acquired pneumonia in ICU	Monotherapy or combination therapy	In-hospital mortality was 49.7%; no clear survival advantage of combination therapy	(Guerci et al., 2019)
Pneumonia	TMP-SMX vs minocycline	Clinical cure: 72.9% vs 66.7%; recurrence was higher with minocycline; infection-related in-hospital mortality did not differ significantly	(Graves et al., 2025)
Pneumonia	Doxycycline or minocycline vs TMP-SMX	Comparable clinical and microbiological outcomes	(Alhayani et al., 2024)
Persistent bloodstream infection in an infant	TMP-SMX initially; switched to cefiderocol monotherapy	Blood cultures cleared within 24 h after cefiderocol	(Hsu et al., 2023)
Nosocomial bloodstream infection	Cefiderocol-based therapy	Clinical success in 62.5%; microbiological eradication in 87.5%; in-hospital mortality 37.5%	(Vena et al., 2025)
Infected pancreatic necrosis	Ceftazidime-avibactam plus aztreonam, with surgical source control	Clinical cure reported	(De Almeida Torres et al., 2023)
Post-neurosurgical meningitis	TMP-SMX plus ciprofloxacin	Successful treatment reported	(Rojas et al., 2009)
Osteomyelitis	TMP-SMX monotherapy	Successful treatment reported	(Osakwe, 2023)
Infective endocarditis	Valve replacement plus levofloxacin and TMP-SMX	Favorable outcome reported	(Qadabashi et al., 2025)

TMP-SMX, trimethoprim-sulfamethoxazole; CZA, ceftazidime-avibactam; ATM, aztreonam. This table summarizes selected observational evidence and should not be interpreted as definitive comparative efficacy data. Treatment should be individualized according to susceptibility testing, infection severity, source control, tolerability, and current guidance.

## 6 Evolution of resistance to key antimicrobial agents and surveillance priorities

### 6.1 Early signals of cefiderocol resistance evolution

Cefiderocol, a novel siderophore cephalosporin, shows promising therapeutic potential against *S. maltophilia* (Nakamura et al., 2021). Interest in this agent has increased in recent years. At the same time, concerns regarding the evolution of resistance to cefiderocol have begun to emerge. Studies have shown that mutations in iron transport–related genes, including *tonB*, *tolQ*, and *cirA*, may contribute to cefiderocol resistance in *S. maltophilia* (Werth et al., 2022). Alterations in iron uptake pathways, together with changes in efflux pump regulatory networks, may influence susceptibility to cefiderocol. Promoter-associated mutations in *smeT* can modify the expression of the SmeDEF efflux system (Werth et al., 2022). As noted in the Introduction, Smc comprises multiple genome-defined lineages. Thus, lineage-associated findings, such as cefiderocol non-susceptibility reported in genogroup 4, should be interpreted as genomic subgroup-specific observations rather than features generalizable to all isolates historically identified as *S. maltophilia* (Sakr et al., 2025). This distinct genetic lineage appears to be associated with a unique genomic background. Cefiderocol offers a promising therapeutic option for *S. maltophilia* infections. However, early signals of resistance evolution warrant close monitoring.

### 6.2 Rising risk of TMP–SMX resistance and key genetic determinants

Resistance to TMP–SMX in *S. maltophilia* has received increasing attention in recent years. According to multicenter surveillance data in China, encompassing 514, 768 clinical isolates collected from 1, 412 medical institutions across 31 provinces, the resistance rate to TMP–SMX decreased from approximately 9.8% in 2014 to 7.5% in 2021 (Wang et al., 2025). Despite this trend, overall resistance levels remain low. Further multicenter surveillance conducted by the China CDC in 2022 reported a TMP–SMX resistance rate of approximately 6.4%, consistent with the longitudinal surveillance findings (Guo et al., 2023). Global trend analyses indicate that resistance to TMP–SMX in *S. maltophilia* shows an upward trend in certain regions, with an average resistance rate of approximately 19.3% reported in Asia (Dadashi et al., 2023). These findings highlight notable regional variability and a potential risk of further increase.

The emergence of TMP-SMX resistance is closely associated with several molecular mechanisms. Studies have shown that class 1 integrons can capture and express resistance genes such as *sul* and *dfr* (Hu et al., 2011). These genes confer resistance to sulfonamides and trimethoprim, respectively, and provide a molecular basis for certain TMP–SMX-resistant phenotypes (Barbolla et al., 2004; Hu et al., 2011). Recent evidence further indicates that mutations in the efflux pump regulatory gene *smeRv* can significantly upregulate the expression of the SmeVWX efflux system, leading to a multidrug-resistant phenotype that includes resistance to TMP–SMX (Boonyong et al., 2025). In addition, overexpression of the SmeDEF efflux pump has also been associated with TMP–SMX resistance (Sánchez and Martínez, 2018). Therefore, continuous surveillance of TMP–SMX resistance and elucidation of its molecular basis are of considerable importance for evaluating its role as a first-line therapy, guiding empirical treatment adjustments, and providing early warning of resistance dissemination.

### 6.3 Expanding trends of resistance to alternative agents

In addition to TMP–SMX and cefiderocol, increasing attention has been directed toward resistance to alternative therapeutic agents such as levofloxacin and minocycline in *S. maltophilia*. Levofloxacin is an important alternative option for the treatment of *S. maltophilia* infections. However, resistance rates among clinical isolates have shown an upward trend in recent years. In certain regions, resistance rates may reach 10%–25% or even higher (Kullar et al., 2022; Banar et al., 2023; Sader et al., 2025). Studies indicate that resistance to levofloxacin is often associated with overexpression of RND efflux pumps (García-León et al., 2014). Biofilm formation can further contribute to reduced susceptibility to levofloxacin in *S. maltophilia* (Río-Chacón et al., 2024). Minocycline, another commonly used alternative agent, generally maintains relatively high susceptibility rates (Sethi et al., 2020; Bostanghadiri et al., 2024). Recent reports, however, indicate a decline in susceptibility, suggesting the emergence of resistant strains. Following the CLSI revision of the minocycline susceptible breakpoint for *S. maltophilia* from ≤4 μg/mL to ≤1 μg/mL, which was introduced in the CLSI 2024 update and retained in CLSI M100-Ed35 (2025), the proportion of isolates categorized as susceptible decreased substantially from approximately 77% to 35% in one analysis (Bakthavatchalam et al., 2024; Clinical and Laboratory Standards Institute, 2025). Resistance to minocycline is largely driven by the overexpression of efflux pump systems. SmeDEF and SmeIJK appear to play particularly important roles in this process (Dadashi et al., 2023). The increasing clinical use of minocycline underscores the importance of further investigating its resistance mechanisms and epidemiological trends. Ongoing surveillance remains essential.

## 7 Emerging non-conventional therapeutic strategies and translational prospects

The complex intrinsic and acquired resistance profile of *S. maltophilia*, together with the limited availability of effective antimicrobial agents, constrains the efficacy of conventional antibiotic therapy (Gil-Gil et al., 2020; Mojica et al., 2022). In response, recent studies have explored non-conventional therapeutic strategies. These approaches have shown promising potential in experimental and early clinical settings. Their translational maturity and clinical feasibility, however, vary considerably and require careful evaluation.

### 7.1 Identification of novel targets and development of new inhibitors ​​

Advances in bioinformatics and systems biology have driven the development of network-based approaches for identifying potential drug targets, which are now an important direction in antimicrobial research. A functional network analysis has identified eight key hub proteins in *S. maltophilia*, including PilL, FliA, Smlt2260, Smlt2267, CheW, Smlt2318, CheZ, and FliM (Pinto et al., 2023). These proteins are mainly linked to motility-, chemotaxis-, and surface-attachment-related processes rather than to direct antimicrobial resistance. PilL is associated with type IV pilus-related functions, whereas FliA and FliM are involved in flagellar regulation and flagellar motor function. Smlt2260 and Smlt2267 are annotated as CheA/CheA2-like chemotaxis sensor kinases, and CheW, CheZ, and Smlt2318 are associated with chemotaxis signal transduction (Pinto et al., 2023). Phytochemicals such as deoxytubulosine and corosolic acid exhibit favorable binding affinities to several of these targets, indicating their potential as alternative antimicrobial agents (Pinto et al., 2023). Nevertheless, these findings are primarily derived from in silico analyses, and their antimicrobial efficacy requires further validation *in vitro* and *in vivo*. Subtractive proteomics approaches have identified several potential therapeutic targets in *S. maltophilia*, including quinone oxidoreductase, dCTP deaminase, phosphotransferase, and the ATP-dependent Clp protease adaptor protein (ClpS). Among these, phosphotransferase and ClpS have emerged as promising novel targets for the development of specific antimicrobial agents (Chakrabarty et al., 2020).

Recent in silico drug repurposing studies have further expanded this strategy by focusing on resistance-associated enzymes. In a 2025 study, Sreenithya et al. screened U.S. Food and Drug Administration (FDA)-approved drugs as potential inhibitors of the chromosomally encoded L1 β-lactamase in *S. maltophilia*. Using molecular docking, molecular dynamics simulation, and MM/PBSA binding free-energy analysis, the authors identified dolutegravir and lumacaftor as promising candidate inhibitors. Dolutegravir showed stronger predicted interaction with L1 β-lactamase than lumacaftor, supporting its potential as a repurposed candidate for combination therapy with β-lactam antibiotics (Sreenithya et al., 2025). Nevertheless, most studies remain confined to computational prediction and network analysis. Consequently, the identified candidate targets and inhibitors still require systematic validation in both *in vitro* and *in vivo* settings.

### 7.2 Efflux pump inhibitors

The development of efflux pump inhibitors (EPIs) capable of suppressing efflux activity is considered a promising therapeutic strategy. Studies have shown that EPIs, such as carbonyl cyanide m-chlorophenylhydrazone(CCCP), phenylalanine–arginine β-naphthylamide (PAβN), and reserpine, can partially restore the susceptibility of *S. maltophilia* to levofloxacin *in vitro* (Zając et al., 2022). Importantly, neither CCCP nor PAβN currently has established clinical utility or sufficient pharmacokinetic and safety data to support therapeutic use for *S. maltophilia* infections. In addition, diclofenac, a non-antibiotic anti-inflammatory drug, has recently been reported to exhibit antibacterial and antibiofilm activity against levofloxacin-resistant *S. maltophilia* isolates. Either alone or in combination with levofloxacin, diclofenac significantly reduced the expression of the efflux pump genes *smeB* and *smeF*. Moreover, the levofloxacin–diclofenac combination markedly decreased the levofloxacin MIC (El-Soudany et al., 2024a). Certain natural compounds, including extracts from Pteris vittata L. and Fallopia japonica, also show potential efflux pump inhibitory activity (Nguyen et al., 2023). These findings suggest their possible role as adjunctive agents in antimicrobial therapy. Most of these findings are derived from *in vitro* screening or mechanistic studies. Their clinical applicability, however, remains to be established.

### 7.3 Anti-virulence interventions and quorum sensing inhibition

QS networks regulate biofilm formation, protease expression, and other virulence-associated phenotypes in *S. maltophilia* (Bhaumik et al., 2023). Consequently, targeting this system has emerged as a promising anti-virulence strategy (Huedo et al., 2018). Recent studies demonstrate that natural compounds, including garlic extracts and cinnamaldehyde, can disrupt QS signaling (Kalia, 2013). This effect inhibits biofilm formation and reduces virulence expression, highlighting their potential as anti-infective agents.

Azithromycin deserves particular attention in this context. Although it is not routinely recommended for treating *S. maltophilia* infections because of intrinsic resistance, it has been reported to exert anti-QS, antibiofilm, and anti-virulence effects. Specifically, azithromycin reduced biofilm formation, protease activity, twitching motility, and the expression of stmPr1, stmPr2, stmPr3, and the QS-related gene *rpfC* in multidrug-resistant *S. maltophilia* clinical isolates (El-Soudany et al., 2024b). In the same study, fenugreek oil, a spice-derived essential oil, also showed anti-virulence activity by reducing protease activity, twitching motility, and the expression of related virulence and QS genes, although its antibiofilm effect was relatively modest (El-Soudany et al., 2024b).

Repurposed non-antibiotic agents have also shown potential in this context. Ascorbic acid has been reported to inhibit biofilm formation and reduce the expression of biofilm-associated genes in *S. maltophilia*, supporting its potential as an antibiofilm adjunct (ElBaradei and Yakout, 2022). Similarly, diclofenac has been reported to exert antibiofilm activity against levofloxacin-resistant *S. maltophilia* isolates, further supporting the potential of repurposed non-antibiotic agents as adjunctive anti-virulence strategies (El-Soudany et al., 2024a). Candidate molecules such as triptolide can also inhibit biofilm formation in *S. maltophilia* and reduce the expression of genes associated with virulence and adaptive phenotypes, including *smeYZ*, *fsnR*, and *bfmAK* (Kim et al., 2018). Most available evidence, however, is derived from *in vitro* studies, and clinical data remain limited (Allen et al., 2014).

### 7.4 Bacteriophage therapy

Bacteriophage therapy has emerged as a promising but still experimental strategy for *S. maltophilia* infections, particularly in the context of multidrug resistance and limited antibiotic options (McCutcheon and Dennis, 2021). Several *S. maltophilia*-targeting phages have been isolated and characterized, showing differences in host range, lifestyle, bactericidal activity, and antibiofilm potential (**Table 5**). A recent case report described personalized phage therapy administered intravenously and through intra-abdominal drains for persistent disseminated *S. maltophilia* infection with pulmonary, bloodstream, and intra-abdominal involvement. Phage therapy was well tolerated and bloodstream clearance was achieved, but intra-abdominal cultures remained positive (Cullen et al., 2024). Therefore, *S. maltophilia* phage therapy should currently be regarded as an investigational or compassionate-use strategy rather than an established clinical treatment.

**Table 5:** Bacteriophages targeting *S. maltophilia* and their characteristics.

Phage name/family	Lifestyle	Tested isolates (%)	Growth inhibition/lytic activity	Biofilm disruption	Reference
DLP1; Siphoviridae	Lytic	8/27 clinical *S. maltophilia* isolates; 29.6%	Not quantified; lytic activity reported	NR	(Peters et al., 2015)
DLP2; Siphoviridae	Lytic	9/27 clinical *S. maltophilia* isolates; 33.3%	Not quantified; lytic activity reported	NR	(Peters et al., 2015)
vB_SmaP_c9-N/Φc9-N; podophage, Autographiviridae	Lytic	NR;tested mainly against strain c24	Effectively inhibited the growth of *S. maltophilia* c24 *in vitro*	Reduced developing and mature biofilms	(Xi et al., 2024)
Smp131; Myoviridae-like, P2-like phage	Temperate	NR; narrow host range reported	Not quantified	NR	(Lee et al., 2014)
ΦSMA5/SMA5; giant myovirus-like phage	Lytic	61/87 *S. maltophilia* strains; 70.1%	Lytic activity reported; burst size ~95 PFU/cell	NR	(Chang et al., 2005)
Yut1; siphovirus-like phage	Lytic	4/4 clinical *S. maltophilia* strains; 100.0%	Strong inhibition in liquid culture at low MOI	NR	(Yamashita et al., 2025)
Yut2; myovirus-like phage	Lytic	3/4 clinical *S. maltophilia* strains; 75.0%	Strongest inhibition among the three Yut phages	NR	(Yamashita et al., 2025)
Yut4; siphovirus-like phage	Lytic	3/4 clinical *S. maltophilia* strains; 75.0%	Moderate inhibition; no resistant bacteria during the 15-h assay	NR	(Yamashita et al., 2025)

NR, not reported; MOI, multiplicity of infection; PFU, plaque-forming units. Family/morphotype is listed according to the descriptions reported in the original studies.

Current evidence for bacteriophage therapy against *S. maltophilia* remains largely limited to *in vitro* and early-stage studies, despite these promising findings. Its clinical application also faces several challenges, including host immune clearance, narrow host specificity, the risk of phage resistance evolution, and the lack of standardized formulations. Further studies are needed to evaluate its safety, efficacy, and long-term stability. This will be essential for advancing its clinical translation.

### 7.5 Nanotechnology: antimicrobial nanoparticles and nanocarrier-based drug delivery systems

Nanotechnology has attracted increasing attention as a potential adjunctive strategy for the treatment of multidrug-resistant *S. maltophilia* infections. This approach mainly includes two aspects: antimicrobial nanoparticles with intrinsic antibacterial activity and nanocarrier-based systems designed to improve the delivery of conventional antimicrobial agents (Mukherjee, 2017; Montoya-Hinojosa et al., 2023). Among antimicrobial nanoparticles, AgNPs are among the most extensively investigated because of their broad-spectrum antimicrobial and antibiofilm activities (Rodrigues et al., 2024). Studies have shown that silver nanoparticles(AgNPs) exhibit significant antimicrobial activity against a variety of multidrug-resistant bacteria, including *S. maltophilia* (Pompilio et al., 2018). These nanoparticles can also disrupt biofilm structures (Pompilio et al., 2018). In addition, extracellular silver nanoparticles biosynthesized by a chromium-reducing strain of *S. maltophilia*, designated OS4, have shown promising antimicrobial activity along with relatively low cytotoxicity (Oves et al., 2013). Other metal-based nanomaterials, including copper-, silver-, and iron-based nanoparticles biosynthesized by *Stenotrophomonas* and *Microbacterium* species, have also been investigated, indicating that antimicrobial nanomaterials beyond AgNPs may have potential applications against bacterial biofilms and resistant pathogens (Mukherjee, 2017).

In addition to nanoparticles with direct antimicrobial activity, nanocarrier-based drug delivery systems may improve the stability, bioavailability, targeted delivery, and local therapeutic performance of antimicrobial agents. Such systems may be particularly useful for biofilm-associated infections, where the extracellular matrix limits antibiotic penetration (Zhang et al., 2022; Yan et al., 2024). For example, curcumin–chitosan nanocomplexes combined with TMP–SMX have been reported to show antimicrobial and antibiofilm activity against clinical isolates of non-fermenting Gram-negative bacteria, including *Stenotrophomonas* isolates. This finding supports the potential of nanocarrier-based systems for delivering conventional antibiotics alone or in combination with antibiofilm compounds (Montoya-Hinojosa et al., 2023). Nevertheless, most available evidence remains limited to *in vitro* studies, and further research is required to evaluate their safety, pharmacokinetics, efficacy, and clinical applicability in *S. maltophilia* infections.

## 8 Discussion and future directions

A major challenge in managing *S. maltophilia* infections lies in its intrinsic resistance and the dynamic interplay between resistance mechanisms and environmental adaptation. Although TMP–SMX remains the first-line agent, its resistance profile varies across clinical settings. The risk of resistance appears to be higher in high-risk populations, particularly ICU patients and those with bloodstream infections (Al-Anazi and Al-Jasser, 2014; Mojica et al., 2022). Minocycline and levofloxacin retain some *in vitro* antimicrobial activity. However, their susceptibility may be compromised by the overexpression of efflux pump systems (García-León et al., 2014; Dadashi et al., 2023; Río-Chacón et al., 2024). These findings indicate that the clinical management of *S. maltophilia* infections should consider the impact of multiple resistance mechanisms on antimicrobial efficacy. This highlights the need for a more integrated treatment approach.

The emergence of new antimicrobial agents has expanded therapeutic options for *S. maltophilia* infections. Cefiderocol shows potent antimicrobial activity against *S. maltophilia*. When used in combination with conventional agents, it may further improve therapeutic efficacy, particularly in moderate-to-severe infections. This supports its potential role in the management of difficult-to-treat cases (Nakamura et al., 2021; Karlowsky et al., 2022; Tamma et al., 2022b; Karakonstantis et al., 2024). Emerging resistance to cefiderocol highlights the need for cautious and closely monitored use. Eravacycline and omadacycline show favorable *in vitro* activity against certain resistant isolates. These agents may therefore represent potential alternative options (Biagi et al., 2020; Morrissey et al., 2020; Xiao et al., 2020; Wu et al., 2023; Al Musawa et al., 2025). Clinical evidence remains limited. This makes it necessary to balance *in vitro* antimicrobial activity with real-world clinical efficacy when considering these agents as alternative options. Meanwhile, combination antimicrobial therapy is receiving increasing attention and may offer additional benefits in this context (Zelenitsky et al., 2005; Church et al., 2013; Hu et al., 2014; Tamma et al., 2022b; Zhao et al., 2022). These strategies remain constrained by the lack of robust large-scale clinical evidence. Their long-term safety profiles also require further validation. This limitation may hinder their broader clinical application.

Non-conventional therapeutic strategies, including QSIs, bacteriophage therapy, and nanotechnology-based drug delivery systems, have demonstrated potential in early-stage research and small clinical studies. These approaches can enhance antimicrobial efficacy by modulating bacterial virulence phenotypes, disrupting biofilm structures, and improving drug delivery. Their clinical translation, however, remains limited by challenges such as formulation stability, targeting specificity, and safety concerns (Chang et al., 2005; Kalia, 2013; Oves et al., 2013; Allen et al., 2014; Lee et al., 2014; Peters et al., 2015; Kim et al., 2018; Pompilio et al., 2018; Chakrabarty et al., 2020; Zając et al., 2022; Nguyen et al., 2023; Pinto et al., 2023; Xi et al., 2024; Yamashita et al., 2025).

Management of *S. maltophilia* infections should be guided by underlying resistance mechanisms, with TMP–SMX remaining the first-line therapy. In high-risk cases or infections caused by resistant strains, alternative agents such as minocycline or fluoroquinolones can be considered, either as monotherapy or in combination. Such approaches may help delay resistance progression and improve clinical outcomes. Future strategies should shift from empirical treatment toward mechanism-based and precision medicine approaches.

## 9 Conclusion

*S. maltophilia* exhibits complex and multifactorial mechanisms of antimicrobial resistance, and increasing resistance to first-line therapies remains a major concern. Key drivers include β-lactamase production, efflux pump overexpression, and biofilm formation. The dissemination of resistance genes via mobile genetic elements further exacerbates this problem. Addressing these challenges requires both optimized combination antimicrobial therapy and the development of novel therapeutic strategies. Future efforts should focus on clarifying resistance regulatory networks, establishing predictive genotype–phenotype models, and accelerating the clinical translation of emerging approaches.
